# Supplementary material for: Nonlinearity synergy: An elegant strategy for realizing high-sensitivity and wide-linear-range pressure sensing
Source: Nat Commun. 2023 Oct 20;14:6641. doi: 10.1038/s41467-023-42361-9 (PMC10589270; doi:10.1038/s41467-023-42361-9)
Supplement: Supplementary file 1 — Supplementary Information [file 41467_2023_42361_MOESM1_ESM.pdf]

Supplementary Information

Nonlinearity synergy: An elegant strategy for realizing high-sensitivity and wide-linear-range pressure sensing

Rui Chen[1], Tao Luo[1], Jincheng Wang[1], Renpeng Wang[1], Chen Zhang[1], Yu Xie[1], Lifeng Qin[1], Haimin Yao[2]*, Wei Zhou[1]*

[1] Pen-Tung Sah Institute of Micro-Nano Science and Technology, Xiamen University, Xiamen, 361102, China

[2] Department of Mechanical Engineering, The Hong Kong Polytechnic University, Hung Hom, Kowloon, Hong Kong SAR, China

* Corresponding author. Email: mmhyao@polyu.edu.hk (H.Y.); weizhou@xmu.edu.cn (W.Z.)

Supplementary Note 1 | Theoretical models for predicting trends in decay constants from different aspect ratios

Since the DPyCF sensing layer is a patterned structure composed of repetitive unit cells (see Supplementary Fig. 7a), the overall electrical resistance of the DPyCF sensing layer is given by $R = R_{\mathrm{uc}}/N$, where R_{uc} represents the resistance of a unit cell and N is the number of the unit cells. Such a proportionality between R and R_{uc} indicates that they must evolve in the same way with the compression strain, or in other words, share the same decay constant (α). Therefore, in the following we only focus on the determination of the decay constant of R_{uc}.

For a unit cell of DPyCF (see Supplementary Fig. 7a), the electrical resistance varies with the compressive strain it undergoes. Due to the symmetry about the middle plane, we just need to consider a half of the unit cell, which is composed of a pyramid and a prismatic foundation of area A_2 and thickness h_2 (Supplementary Fig. 7b). To avoid the computational complexity involving the tapering pyramid, we further simplify it by a prism with a smaller cross sectional area of A_1 and height of h_1 (see Supplementary Fig. 7c). The aspect ratio of the DPyCF structure can be roughly correlated with A_1 and h_1 through $\lambda \cong h_1/\sqrt{A_1}$.

Under a compressive force *F* (see Supplementary Fig. 7d), the unit cell contracts by:

$$\Delta = \delta h_1 + \delta h_2 \cong 2\left(\frac{Fh_1}{EA_1} + \frac{Fh_2}{EA_2}\right) \tag{S1}$$

where *E* is the effective elastic modulus of the DPyCF (porous carbon foam). The average compressive strain of the unit cell is given by:

$$\bar{\varepsilon} = \frac{\Delta}{2(h_1 + h_2)} = \frac{F(A_1h_2 + A_2h_1)}{EA_1A_2(h_1 + h_2)} \tag{S2}$$

Based on the Eqs. (S1) to (S2), the compressive strains in two prisms can be expressed in terms of the effective strain $\bar{\varepsilon}$ as:

$$\varepsilon_1 = \frac{\Delta h_1}{h_1} = \frac{A_2(h_1 + h_2)}{A_1 h_2 + A_2 h_1}\bar{\varepsilon} = \frac{A_2(h_1 + h_2)\lambda^2}{A_2\lambda^2 h_1 + h_1^2 h_2}\bar{\varepsilon}$$
$$\varepsilon_2 = \frac{\Delta h_2}{h_2} = \frac{A_1(h_1 + h_2)}{A_1 h_2 + A_2 h_1}\bar{\varepsilon} = \frac{(h_1 + h_2)h_1}{h_1 h_2 + A_2\lambda^2}\bar{\varepsilon} \quad \text{(S3)}$$

Here the relationship $\lambda \cong h_1/\sqrt{A_1}$ is applied to replace A_1. For porous conductive material, the electrical resistivity exhibits a strong dependence on the compressive strain it is subjected. Such a strain-dependent resistivity is mainly attributed to the increase of the conductive points under compression, and can be expressed by (*J. Mater. Sci.*, 2011, 46, 3186-3190):

$$\rho = \rho_0 \exp(-\varepsilon/\varepsilon_0) \quad \text{(S4)}$$

where ρ_0 is the electrical resistivity at zero strain state and ε_0 is a characteristic strain. For our sample (porous carbon foam), it is determined through experimental measurement and curve fitting that $\rho_0 = 4000\ \Omega\text{m}$ and $\varepsilon_0 = 0.3$.

$$R_{\text{uc}} = 2\left[\frac{\rho_1 h_1(1 - \varepsilon_1)}{A_1} + \frac{\rho_2 h_2(1 - \varepsilon_2)}{A_2}\right] \quad \text{(S5)}$$

where $\rho_1 = \rho_0 \exp(-\varepsilon_1/\varepsilon_0)$ and $\rho_2 = \rho_0 \exp(-\varepsilon_2/\varepsilon_0)$ are the resistivity of two compressed prisms.

The electrical resistance of the unit cell can be expressed in terms of the average strain ($\bar{\varepsilon}$) then is given by:

$$R_{\mathrm{uc}} = 2\rho_0 \exp\left(-\frac{A_2\left(\lambda\sqrt{A_1}+h_2\right)\bar{\varepsilon}}{\varepsilon_0\left(A_2\lambda\sqrt{A_1}+A_1h_2\right)}\right)\left(1-\frac{A_2\left(\lambda\sqrt{A_1}+h_2\right)}{A_2\lambda\sqrt{A_1}+A_1h_2}\bar{\varepsilon}\right)\frac{\lambda}{\sqrt{A_1}}$$

$$+2\rho_0 \exp\left(\frac{-\bar{\varepsilon}(\lambda\sqrt{A_1}+h_2)\sqrt{A_1}}{\varepsilon_0(A_2\lambda+h_2\sqrt{A_1})}\right)\left(1-\frac{(\lambda\sqrt{A_1}+h_2)\sqrt{A_1}}{A_2\lambda+h_2\sqrt{A_1}}\bar{\varepsilon}\right)\frac{h_2}{A_2} \quad \text{(S6)}$$

Take $h_2 = 0.5$ mm, $A_1 = 0.25$ mm^2, $A_2 = 1$ mm^2, the variation of R_{uc} with the average strain $\bar{\varepsilon}$ as given by Eq. (S6) is shown in Supplementary Fig. 8 for different aspect ratio $\lambda = 0.25, 0.5, 1,$ and 2. In all cases, R_{uc} exhibits a decaying behaviour with increasing $\bar{\varepsilon}$, which can be perfectly fitted by an exponential function $R_{\mathrm{uc}}(\bar{\varepsilon}) = R_{\mathrm{uc}}^0 \exp(-\bar{\varepsilon}/\alpha)$, where R_{uc}^0 stands for the resistance at zero strain and α is the decay constant. The dependence of the decay constant (α) on the aspect ratio (λ) is shown in Supplementary Fig. 9, which agrees well with the decay constant of the DPyCF sensing layer obtained by experimental measurement and curve fitting (see Supplementary Fig. 6).

Supplementary Note 2 | Robot grasping closed-loop control algorithm

A closed-loop algorithm, which uses a pressure-based stiffness estimator to achieve an adaptive regulation of the grasping force, is applied to control the robotic gripper. A wavelet transform-based slip detection algorithm was used to detect the slip. The detailed flow of the closed-loop algorithm is described below:

1. The robotic gripper grasps the object, and the pressure sensor detects the pressure applied to the object simultaneously.

2. The pressure signals are processed by the stiffness estimator to calculate the initial grasping force.

3. The robot attempts to lift the object with the calculated grasping force and the pressure, which is monitored and analyzed by the slip detection algorithm. A wavelet transform-based algorithm for slip detection was used to obtain the detail coefficient, which is the high-frequency information of the pressure signal. When the obtained detail coefficient exceeds a threshold that is determined based on the grasping force, it is judged as a slip by the algorithm.

4. If the slip is detected, the robotic gripper was moved to its initial position and a larger grasp force is applied for a new grasping-and-lifting trial. The increment of the grasping force is calculated based on the object's stiffness and sliding speed.

5. If no slip is detected, the robot holds the object and keeps the lifted status for 10 seconds before lowering and releasing the object, and the whole program ends.

Supplementary Note 3 | Applicability of strategy of nonlinearity synergy in capacitive sensors

To verify the applicability of our strategy of nonlinearity synergy in the other types of pressure sensors, we designed and fabricated capacitive sensors by using carbon nanotubes (CNTs)-doped polydimethylsiloxane (PDMS) as the material for pressure sensing layers. The sensing layer is prepared by assembling two pieces of PDMS/CNT sheets with micro-pyramid array on one side in a face-to-face configuration (Supplementary Fig. 21). Then, the as-prepared sensing layer is sandwiched between a pair of electrodes, forming a capacitive pressure sensor. To modulate the capacitive and mechanical properties of the sensing layers, we adopted two different mass factions of CNTs, 5% and 3%, when preparing the sensing layers. The resultant sensors are named as CNT@5 and CNT@3, respectively.

The capacitances of these two kinds of sensors were tested under a varying compressive strain (ε) (Supplementary Figs. 22a and 22d), showing ever-increasing capacitances (C) of the sensors with the applied compressive strain (ε). The strain dependence of the capacitances can be perfectly ($R^2 >$ 0.99) fitted by exponential functions in the form of

$$C = C_0 \exp(\varepsilon/\gamma) \quad \text{(S7)}$$

where C_0 is the initial capacitance at zero strain and γ is a constant characterizing the increasing rate of the capacitance with the compressive strain. For CNT@5 and CNT@3, we found $\gamma_1 = 0.459$ and $\gamma_2 = 2.357$ (Supplementary Figs. 22a and 22d), respectively. From Eq. (S7), the relative variation of the capacitance ($\Delta C/C_0$) thus can be expressed as a function of ε:

$$\frac{\Delta C}{C_0} = \frac{C - C_0}{C_0} = \exp(\varepsilon/\gamma) - 1 \quad \text{(S8)}$$

On the other hand, the mechanical behaviour of CNT@5 and CNT@3 under compression was also characterized (Supplementary Figs. 22b and 22e). The nominal pressure (p), which is defined as the applied force divided by the area enclosed by the outer perimeter of the sensor, exhibits a clear nonlinear dependence on the compressive strain (ε). Such nonlinear pressure-strain relationships can be perfectly (R^2 > 0.99) fitted by an exponential function as:

$$p = \beta E_0[\exp(\varepsilon/\beta) - 1] \quad \text{(S9)}$$

where E_0 is the initial tangential modulus of the sensor at zero strain and β is the stiffening constant. For CNT@5 and CNT@3, we found $\beta_1 = 0.314$ and $\beta_2 = 0.235$, respectively (Supplementary Figs. 22b and 22e). In the light of Eqs. (S8) and (S9), the sensitivity (S) of the sensor can be expressed as:

$$S \equiv \frac{d(\Delta C/C_0)}{dp} = \frac{1}{\beta E_0} \exp\left[\left(\frac{1}{\gamma} - \frac{1}{\beta}\right)\varepsilon\right] \quad \text{(S10)}$$

Eq. (S10) implies that the linearity of the capacitive sensor depends on the difference between γ and β, which is similar to what is implied in Eq. (4) for the DPyCF@SR resistive sensors. Ideally, perfect linearity (R^2 = 1.0) can be achieved when $\beta = \gamma$. In practice, however, γ and β would not be exactly the same. Under this circumstance, the closer the values of γ and β, the higher the linearity of the sensors. This theoretical prediction from Eq. (S10) was verified by comparing the linearity of CNT@5 and CNT@3. For CNT@5 with $\beta_1 = 0.314$ and $\gamma_1 = 0.459$, a high linearity with R^2 > 0.99 was measured in the range of 0-100 kPa (Supplementary Fig. 22c). In contrast, for CNT@3 with $\beta_2 = 0.235$ and $\gamma_2 = 2.357$, a relatively lower linearity (R^2 < 0.95) was detected (Supplementary Fig. 22f). It is demonstrated that our strategy of nonlinearity synergy can also be applied to the capacitive pressure sensors for a wider linearity range.

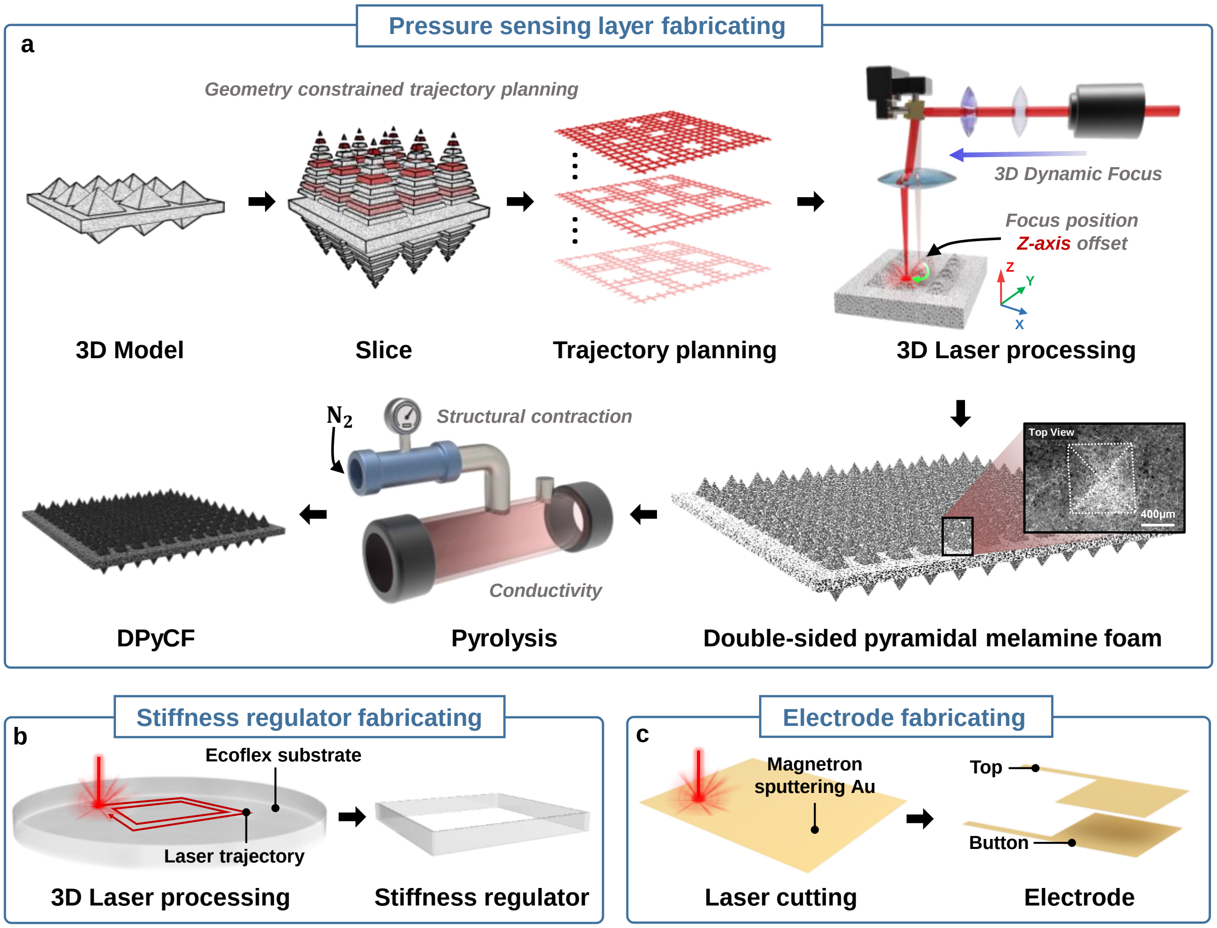

Supplementary Fig. 1 | Fabrication processes of the DPyCF sensor. a Fabrication processes of the sensing layer. Firstly, a 3D model of the pyramidal array is created with CAD software (SoildWorks), and then the 3D model is sliced for trajectory planning of the laser processing. Subsequently, a planned processing trajectory is introduced into an infrared picosecond laser, and then the melamine foam (MF) is manufactured using the 3D dynamic focusing technique of the laser to obtain an MF-based double-sided pyramidal array. Finally, the MF-based double-sided pyramidal array is pyrolyzed at a high temperature to produce conductive double-sided pyramidal carbon foam (DPyCF). **b** Fabrication processes of the stiffness regulator. **c** Fabrication processes of the electrode.

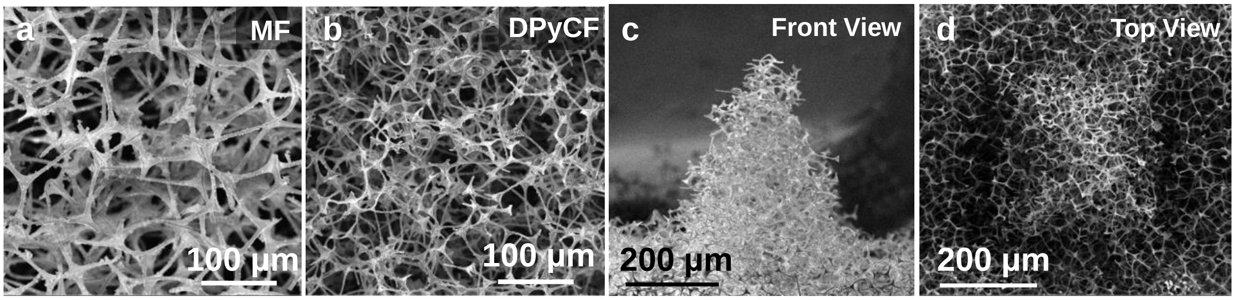

Supplementary Fig. 2丨Scanning electron microscopy of the MF before and after pyrolysis. **a and b** SEM images of MF and DPyCF. The SEM images reveal that the MF skeleton shrinks significantly after pyrolysis. **c and d** SEM images of the front view and top view of DPyCF showing the porous pyramidal structure.

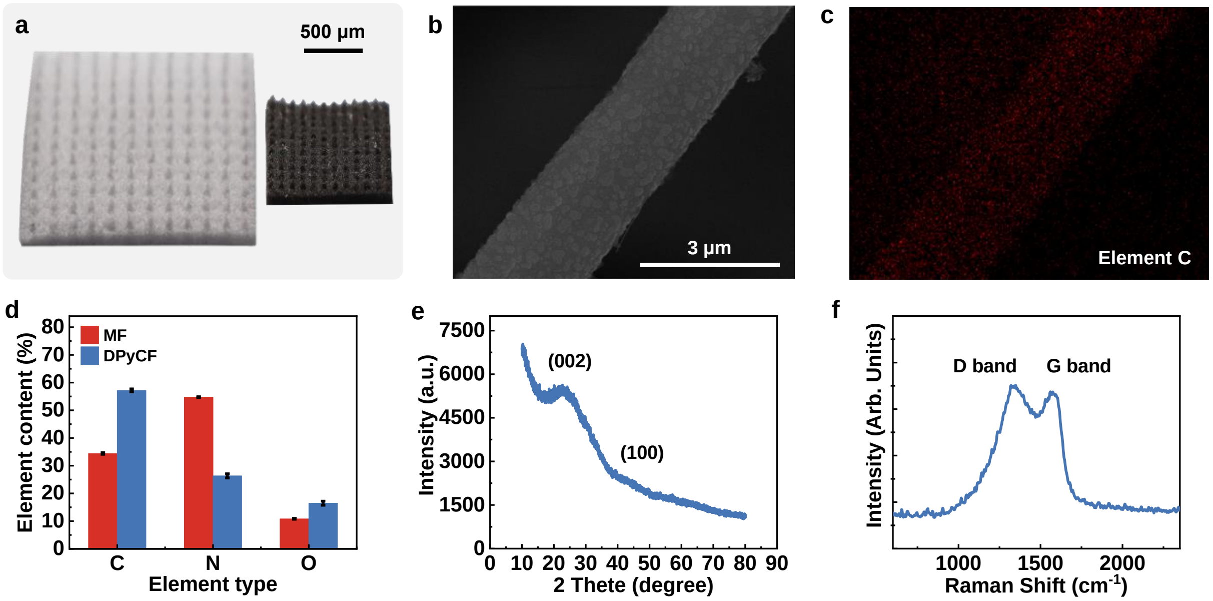

Supplementary Fig. 3 | Material characterization of the MF pyramidal array and DPyCF array. a Optical image of MF pyramidal array and DPyCF array. The images show that the DPyCF volume reduced significantly (~90 %). After the pyrolysis process, the bottom width of the pyramids decreased from approximately 800 μm to 400 μm, while the height reduced from approximately 1 mm to 500 μm. **b** An SEM image of the DPyCF. **c** The EDS elemental analysis result of the DPyCF. **d** C, N, and O elemental content of MF and DPyCF. The results reveal a significant increase in C elements and a significant decrease in N elements for DPyCF (n = 3 samples; center, mean; error bars, s.d.). **e** XRD analysis of the DPyCF. **f** Raman spectroscopy of the DPyCF.

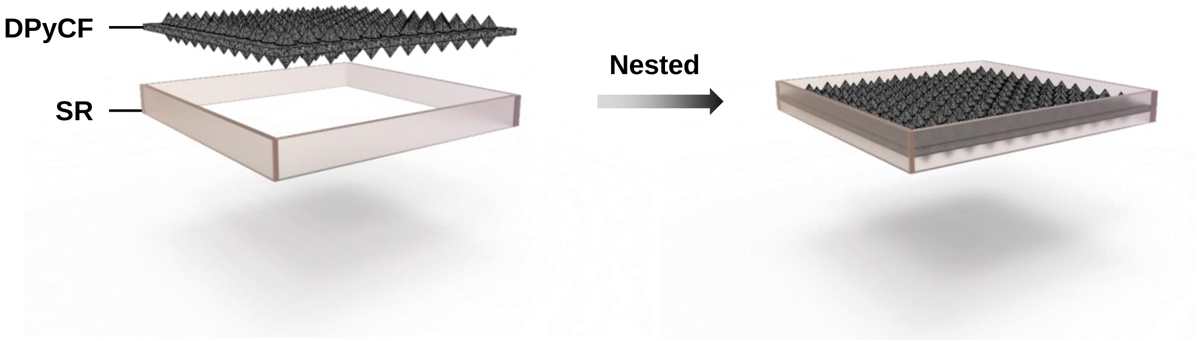

Supplementary Fig. 4 | The schematics of SR and DPyCF nested. The fabricated sensing layer (DPyCF) is nested inside the SR (outside dimensions 9 × 9 mm^2, inside dimensions 7 × 7 mm^2) along the vertical direction, which ensures that the positive pressure along the vertical direction can be uniformly applied to the surface of the sensor layer and SR.

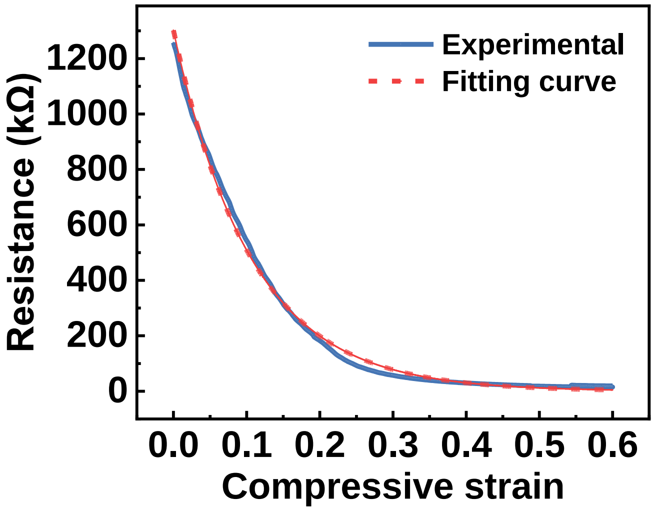

Supplementary Fig. 5 | The variation of electrical resistance of a double-sided pyramidal carbon foam (DPyCF) with the compressive strain. The fitting result ($R^2 = 0.996$) can be described as: $R = R_0 \exp(-\varepsilon/\alpha)$, where the resistance at zero strain $R_0 = 1302$ kΩ and the decay constant $\alpha = 0.11$.

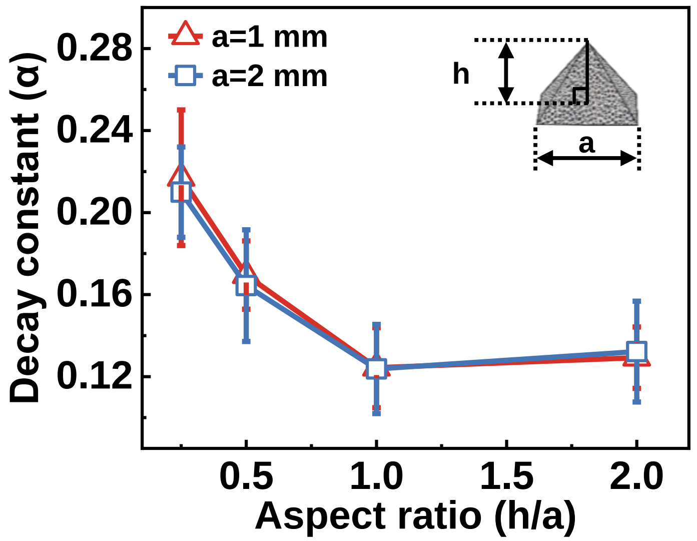

Supplementary Fig. 6 | The dependence of the decay constant (α) on the aspect ratio (height over base) of the micro-pyramid (n = 6 samples; center, mean; error bars, s.d.). As the aspect ratio grows, the decay constant (α) tends to drop and then gradually increase. Different bottom-of-the-pyramid widths have minimal influence on the change of the attenuation constant.

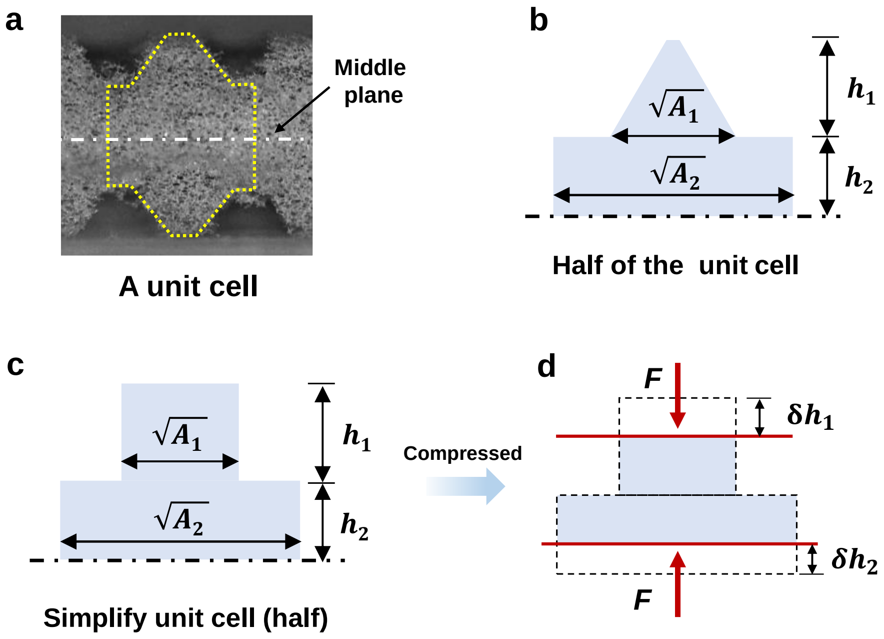

Supplementary Fig. 7 | Schematic of the theoretical model for revealing the effect of DPyCF aspect ratio on the decay constant of the electrical resistance. a The cross-sectional image of a DPyCF sensing layer showing the patterned structure composed of repetitive unit cells. **b** A half of the unit cell with a pyramid and a prismatic foundation of area A_2 and thickness h_2. **c** Schematic diagram of the simplified model. **d** Schematic diagram showing the deformed configuration of the model under compression by a force F.

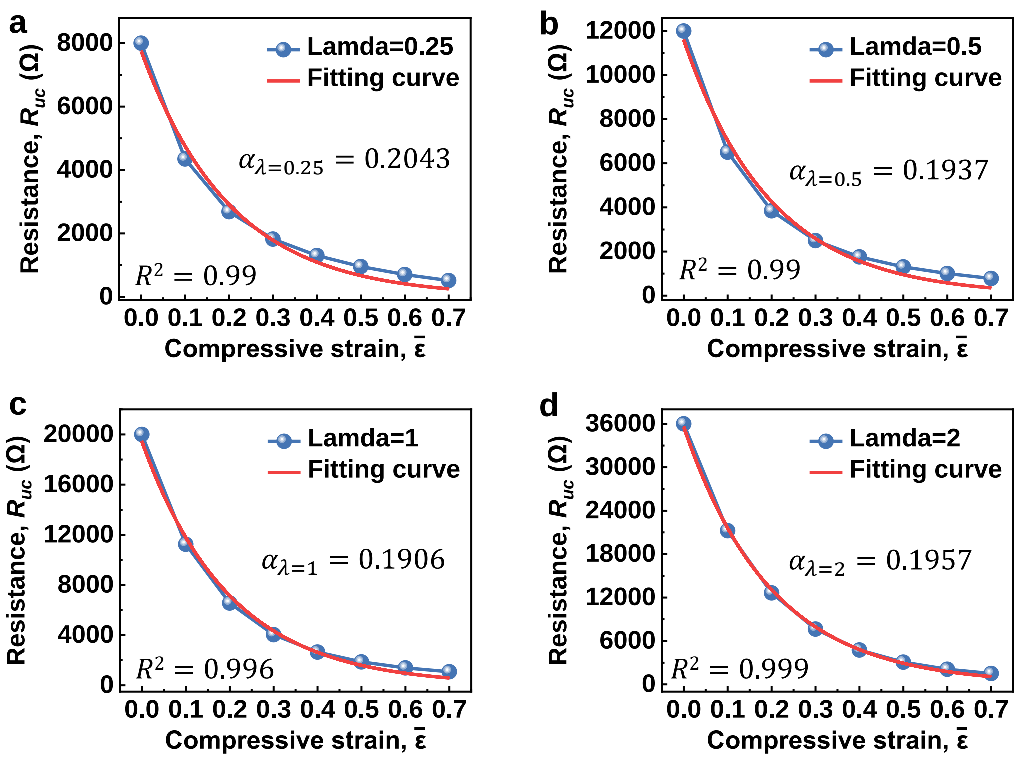

Supplementary Fig. 8 | Variations of the theoretically predicated electrical resistance of a unit cell (R_{uc}) with the subjected compressive strain ($\bar{\varepsilon}$) for DPyCF of different aspect ratios (λ). Such decay of the resistance with the compressive strain is fitted with the an exponential function $R_{\mathrm{uc}} = R_{\mathrm{uc}}^{0} \exp(-\bar{\varepsilon}/\alpha)$, where the fitting parameter α is called decay constant. a λ = 0.25, **b** $\boldsymbol{\lambda}$ = 0.5 **c** $\boldsymbol{\lambda}$ = 1, and **d** $\boldsymbol{\lambda}$ = 2.

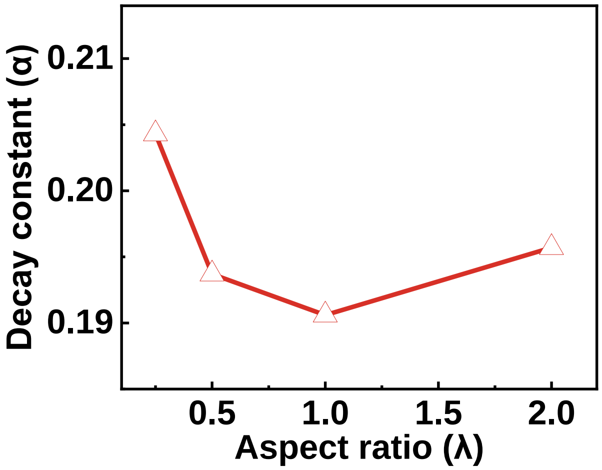

Supplementary Fig. 9 | Theoretically predicted dependence of the decay constant (α) on the aspect ratio (λ) of the DPyCF structure. The theoretical predicted results of the decay constant (α) first decreases and then gradually increases as the aspect ratio increases, which is consistent with the experimental results (Supplementary Fig. 6).

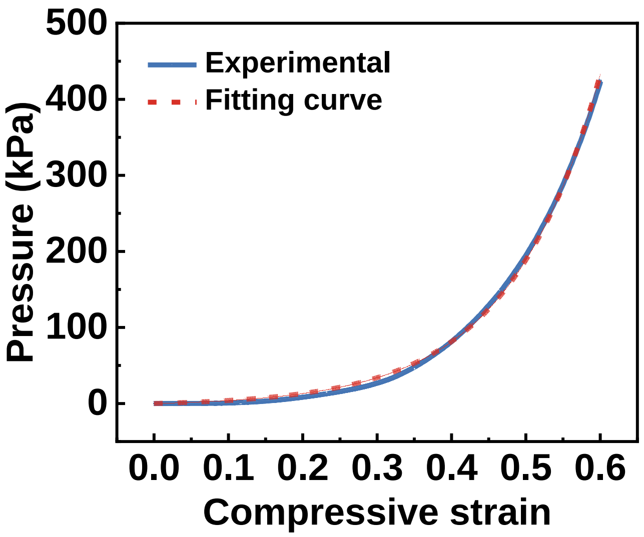

Supplementary Fig. 10 | The measured compressive force-strain curve of the ensemble of SR and the sensing layer (DPyCF). The fitting result ($R^2 = 0.998$) can be described as: $p = E_0\beta[\exp(\varepsilon/\beta) - 1]$, where the tangential modulus at zero strain $E_0 = 26.09$ kPa and the stiffening constant $\beta = 0.122$.

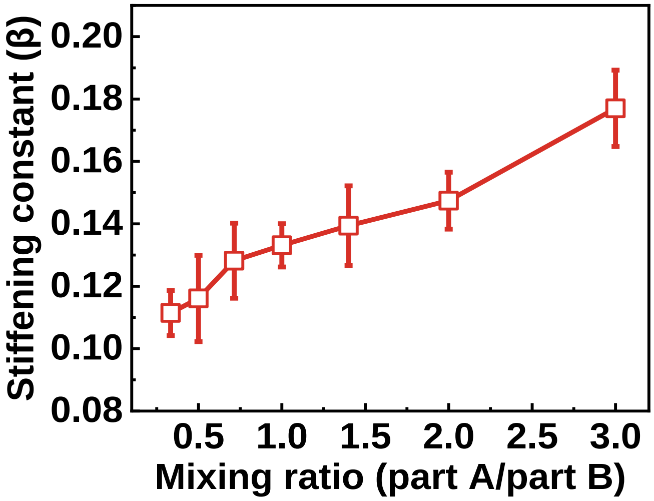

Supplementary Fig. 11 | The dependence of the stiffening constant (β) on the mixing ratio of two compositions in the Ecoflex rubber (n = 6 samples; center, mean; error bars, s.d.). As the mixing ratio (part A/ part B) increases, the stiffening constant (β) gradually increases.

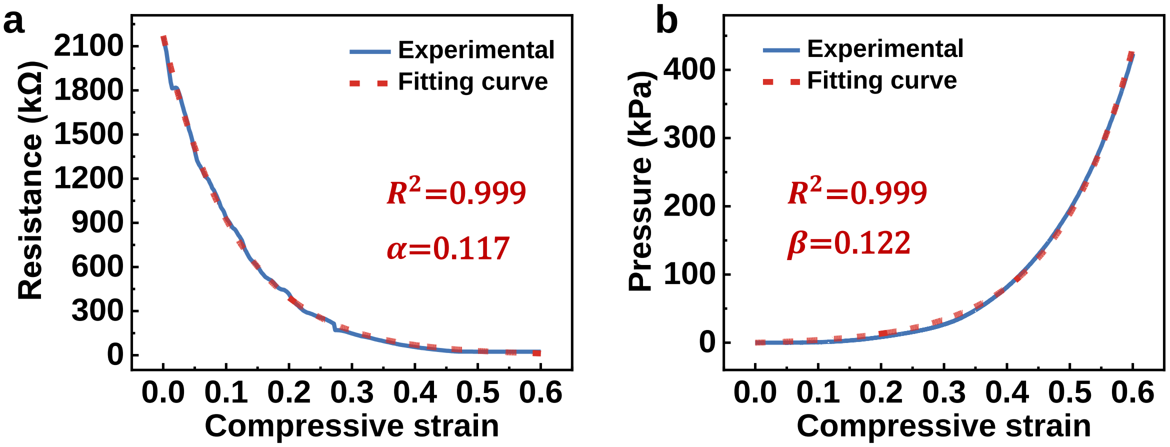

Supplementary Fig. 12 | A prototype of a well-designed DPyCF@SR pressure sensor. a The variation of electrical resistance of a double-sided pyramidal carbon foam (DPyCF) with the compressive strain (α = 0.117). **b** The measured compressive force-strain curve of the ensemble of SR and the sensing layer (β = 0.122).

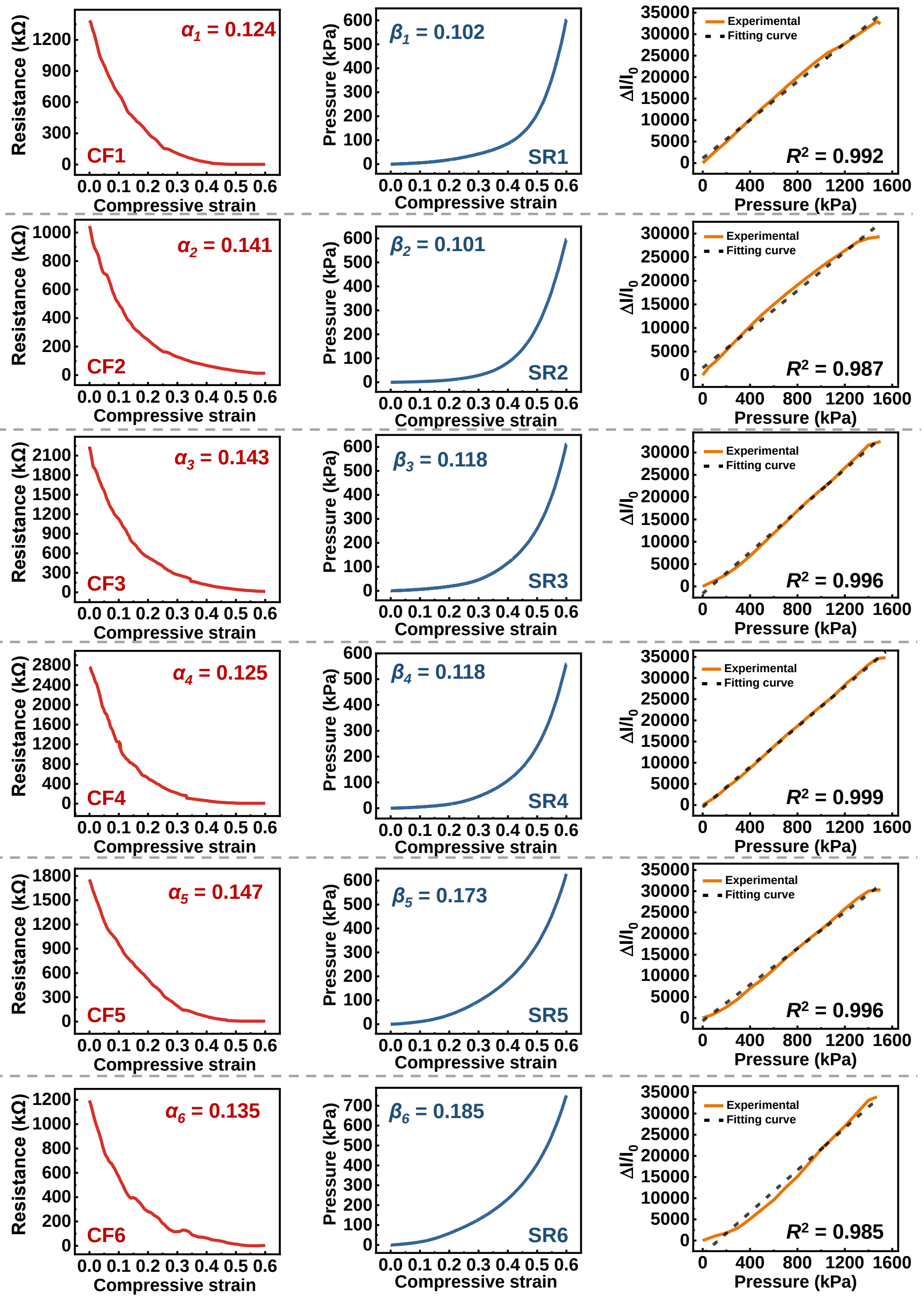

Supplementary Fig. 13 | Six DPyCF@SR sensors with different combinations of decay constant (α) and stiffening constant (β). The decay constant (α) of the six sensors were 0.124, 0.141, 0.143, 0.125, 0.147, and 0.135, the stiffening constant (β) were 0.102, 0.101, 0.118, 0.118,

0.173, and 0.185, and the linearity of the tested sensors' coefficient of determination were 0.992, 0.987, 0.996, 0.999, 0.996, and 0.985 respectively.

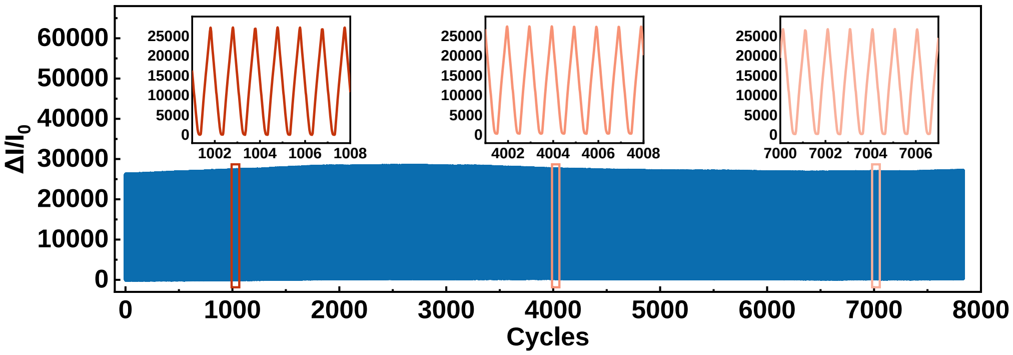

Supplementary Fig. 14 | Output (relative change of current) of a DPyCF@SR sensor under a cyclic pressure load with amplitude of 1 MPa and approximately 7,800 cycles. The DPyCF@SR sensor can still output an undamped electrical signal, which shows higher signal stability.

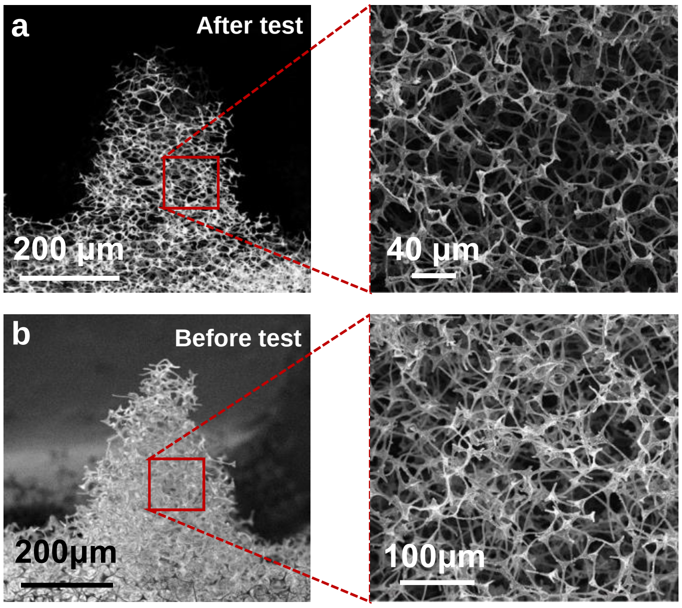

Supplementary Fig. 15 | Scanning electron microscopy (SEM) of the DPyCF before and after high-pressure cyclic compression. a SEM images of the DPyCF after high-pressure (1 MPa) cyclic compression. **b** SEM images of the DPyCF before the compression test.

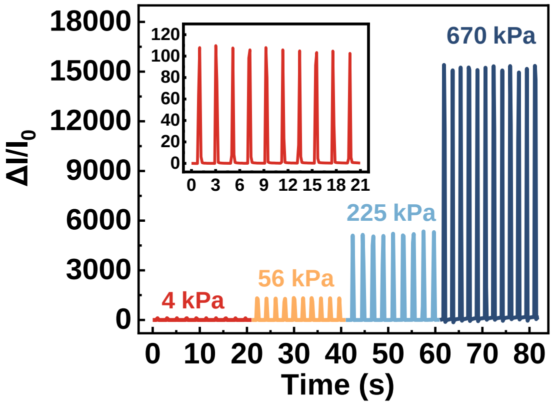

Supplementary Fig. 16 | The current response to cyclic pressures of 4, 56, 225, and 670 kPa. This experiment regulates the amount of pressure by varying the descending distances of the loading device.

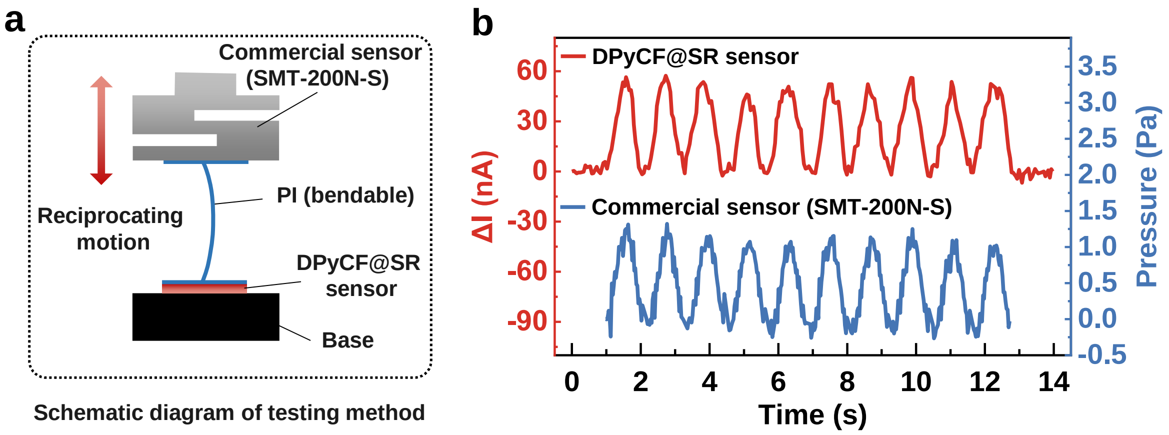

Supplementary Fig. 17 | Ultra-low cyclic pressure test. a Schematic diagram showing the setup for ultra-low cyclic pressure test. The force causing the bending of the polyimide (PI) film is equally applied to the DPyCF@SR sensor and the commercial high-precision force sensor (SMT-200N-S, AiLogics, USA). **b** The output (current change) of the DPyCF@SR sensor agrees well with the output of the commercial force sensor, reflecting the high performance of the DPyCF@SR sensor in detecting cyclic lower pressure.

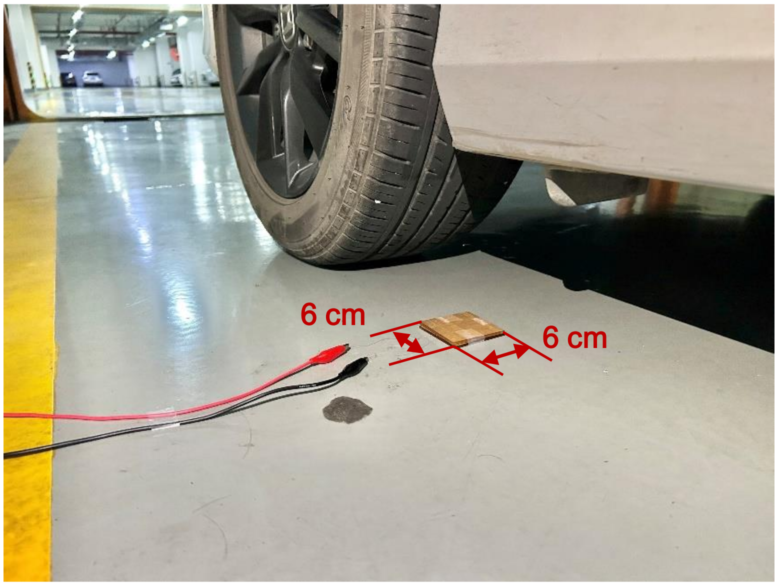

Supplementary Fig. 18 | Experimental setup for detecting the variation of load weight in a sedan weighting ~1.5 tons. The sensor is permanently installed in the center of two 6 cm square acrylic panels.

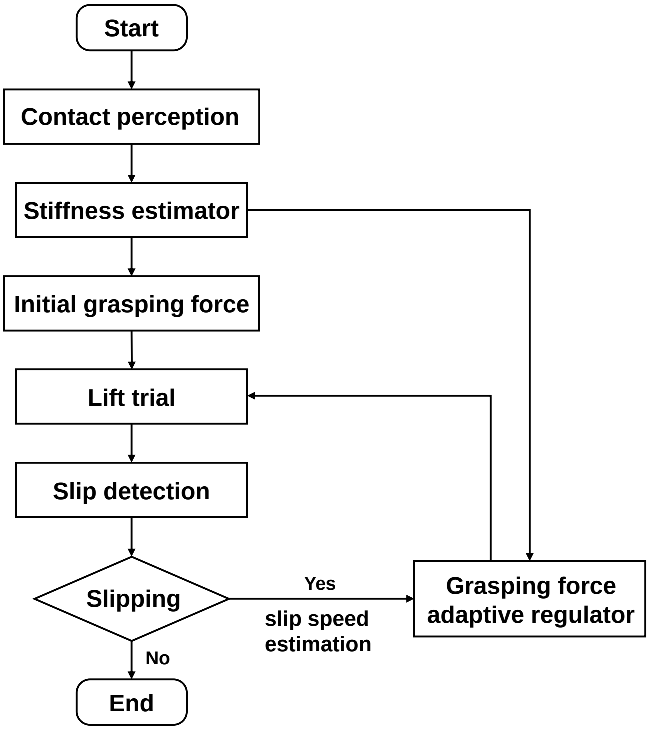

Supplementary Fig. 19 | Flow chart of the robotic grasping-and-lifting operation. The closed-loop control algorithm can automatically adjust the size of the grasping force to realize the adaptive grasping of objects with different stiffness.

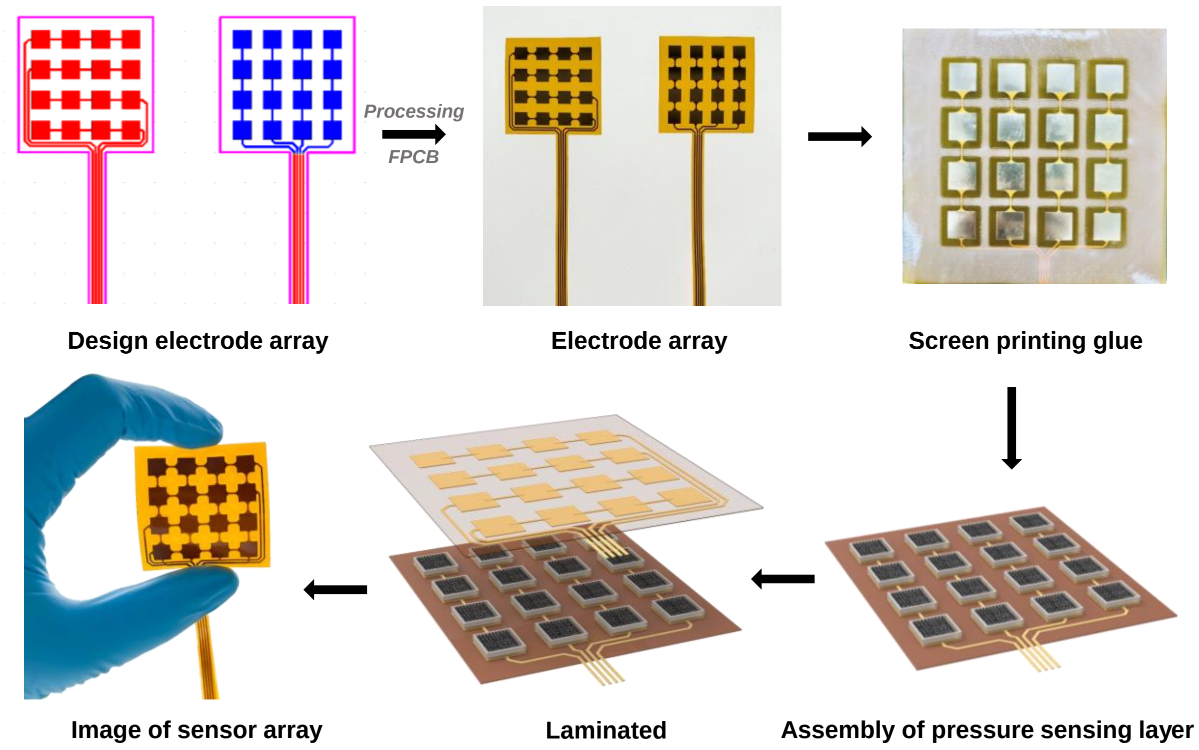

Supplementary Fig. 20 | Fabrication processes of the DPyCF@SR sensor array. First, flexible circuits are designed using electronic design automation (EDA) software. Next, the designed circuits are fabricated into flexible circuit boards (FPCBs) through machining processes. Subsequently, screen printing is conducted on the surface of the FPCB, and glue is applied to the electrode side of the FPCB. The machined DPyCF and SR components are then assembled onto the FPCB electrodes. Finally, another FPCB is tightly assembled face-to-face with the FPCB containing the pressure-sensitive layer, completing the fabrication of the DPyCF sensor array.

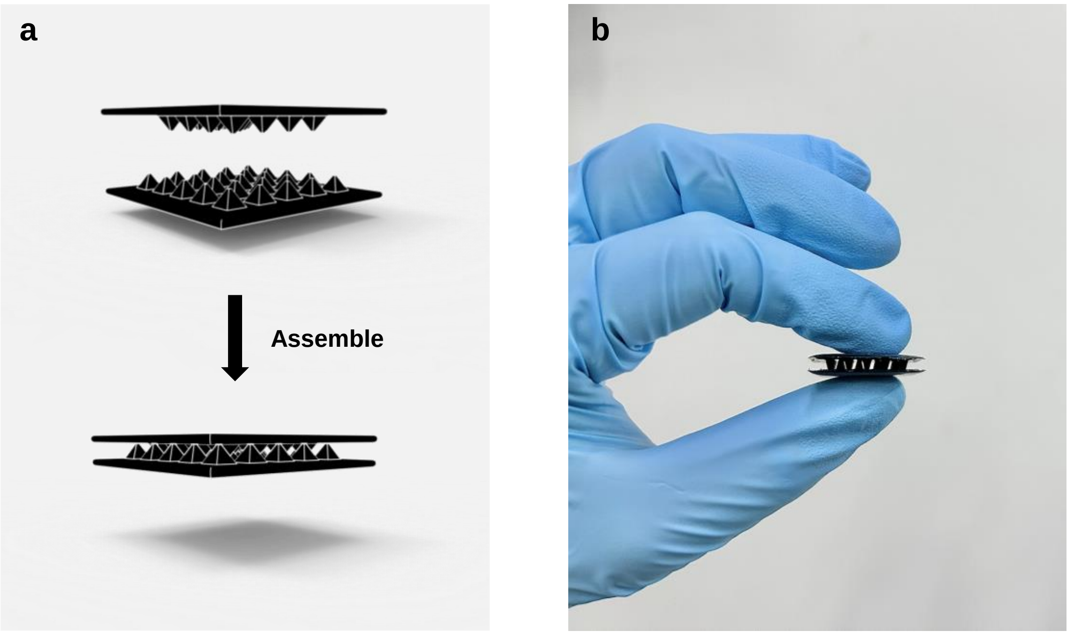

Supplementary Fig. 21 | Capacitive pressure sensor made of polydimethylsiloxane (PDMS) doped with carbon nanotubes (CNTs). a The schematic diagram showing the design of the capacitive sensor. **b** The photograph of an as-prepared capacitive pressure sensor.

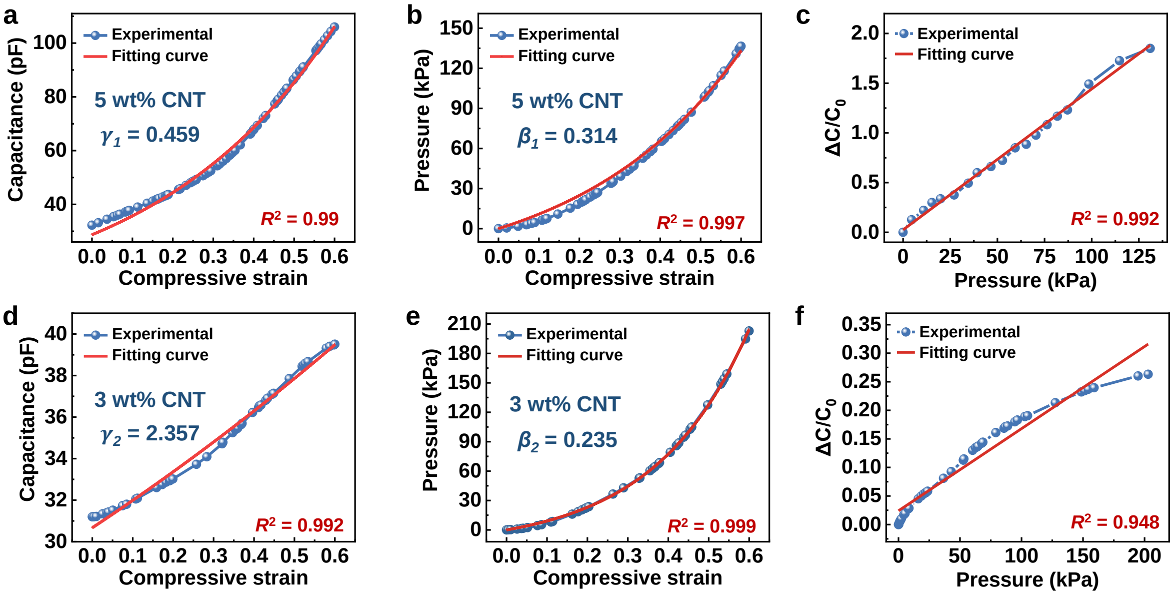

Supplementary Fig. 22 | Characterizations of two capacitive pressure sensors. a The variation of capacitance of CNT@5 with varying compressive strain. **b** The compressive pressure-strain curve of the CNT@5. **c** Relative capacitance changes as a function of pressure for the CNT@5. **d** The variation of capacitance of CNT@3 with varying compressive strain. **e** The compressive pressure-strain curve of the CNT@3. **f** Relative capacitance changes as a function of pressure for the CNT@3.

Supplementary Table 1 | Comparison of our work with representative reported works.

No.	Sensitivity (kPa^{-1})	Range (kPa)	Linearity (Y/N)	Response time (ms)	Number of cycles (times)	Mechanisms	Fabrication process (method adopted)	Ref.
1	41 (0-0.4 kPa)<br>13 (0.4-5 kPa)<br>10 (5-10 kPa)<br>5.2 (10-15 kPa)<br>2.1 (15-50 kPa)	50	No	20	5000	Capacitive	1. Photolithography<br>2. Template method<br>3. O_2 plasma treatment<br>4. Anneal	15
2	30.11 (0-10 kPa)<br>8.42 (10-40 kPa)<br>1.03 (40-115 kPa)	115	No	29/37	5000	Capacitive	1. Template method<br>2. O_2 plasma treatment<br>3. Laser process	16
3	3.13 (0-1 kPa)<br>1.65 (1-5 kPa)<br>1.16 (5-10 kPa)<br>0.68 (10-30 kPa)<br>0.43 (30-50 kPa)	50	No	94	5000	Capacitive	1. Immersion dissolution method<br>2. Ultra sonication	9
4	0.314 (0-1000 kPa)	1000	Yes	N/A	5000	Capacitive	1. Milling<br>2. Template method<br>3. Magnetic field assisted processing	23
5	0.15 (0-47 kPa)<br>0.08 (47-214 kPa)<br>0.04 (214-450 kPa)	450	No	6	10000	Capacitive	1. Template method<br>2. O_2 plasma treatment<br>3. Chemical interlinking	24
6	8.5 (0-12kPa)	12	Yes	40	10000	Piezoresistive	1. Photolithography<br>2. Selective wet etching<br>3. Template method<br>4. CVD method	10
7	32.42 (0-0.2 kPa)<br>8.03 (0.2-1 kPa)	1	No	40	8000	Piezoresistive	1. Textile process	17
8	50.9 (0-1 kPa)	20	No	50	20000	Piezoresistive	1. Chemical synthesis	18

	18.96 (1-20 kPa)						2. Blade-coating method	
9	4.52 (0-3 kPa)<br>28.34 (3-10 kPa)	10	No	87	6000	Piezoresistive	1. Ink-jet printing<br>2. Magnetron sputtering<br>3. Electrospinning method<br>4. Template method<br>5. Thermal reduction method	19
10	44.5 (0-1.2 kPa)	1.2	Yes	N/A	5500	Piezoresistive	1. CVD method<br>2. Electrospinning method<br>3. Anneal<br>4. Membrane transfer method	20
11	11.4 (0-3.5 kPa)<br>4.8 (3.5-10 kPa)<br>1.84 (10-40 kPa)	40	No	47	1000	Piezoresistive	1. Sacrificial powder template method<br>2. Spray-coating method	11
12	4.68 (0-150 kPa)<br>11.09 (150-200 kPa)	200	No	N/A	1000	Piezoresistive	1. Laser reduction method	25
13	17.5 (0-120 kPa)	120	Yes	90/220	6000	Piezoresistive	1. Oxidation polymerization method<br>2. Spray-coating process	21
14	3.5 (0-218 kPa)	218	Yes	21/52	9000	Piezoresistive	1. Chemical synthesis<br>2. Template method<br>3. Blade-coating method<br>4. Sputter	26
15	6.4 (0-800 kPa)	800	Yes	N/A	2000	Piezoresistive	1. Laser process<br>2. Template method<br>3. Spray-coating process	27
16	5.61 (0-220 kPa)<br>2.2 (220-600 kPa)	600	No	60	33000	Piezoresistive	1. Template method<br>2. Dip-coating method<br>3. Etched method	28
17	5.3 (0-1.3 kPa)	160	No	50/20	1000	Piezoresistive	1. Chemical synthesis	12

	2.27 (1.3-10.2 kPa)<br>0.57 (10.2-40.7 kPa)<br>0.08 (40-160 kPa)						2. Dip-coating method	
18	5 (0-5 kPa)<br>1 (5-50 kPa)<br>0.005 (50-1000 kPa)	1000	No	8	4000	Piezoresistive	1. Chemical synthesis<br>2. Freeze-drying process	29
19	8.3 (0-10kPa)<br>4.3 (10-30kPa)<br>1.5 (30-200kPa)	200	No	60/70	10000	Piezoresistive	1. Laser process<br>2. Template method<br>3. Spray-coating process	30
20	0.9 (0-0.6 kPa)<br>11.06 (0.6-10 kPa)<br>4.5 (10-30 kPa)	30	No	N/A	1000	Piezoresistive	1. Laser process	13
21	10.805 (0-1 kPa)<br>2.015 (1-10 kPa)	10	No	N/A	1000	Piezoresistive	1. Mechanical force method<br>2. Sacrificial powder template method	14
22	4.7 (0-1000 kPa)	1000	Yes	24/15	35000	Piezoresistive	1. Direct ink writing printing<br>2. Powder sacrifice method	31
Our work	**24.6 (0-1400 kPa)**	**1400**	**Yes**	8.4/9.2	50000	Piezoresistive	1. Laser process<br>2. Pyrolysis	

Supplementary Table 2 | Theoretically calculated linearities of sensors with different combinations of the decay constant (α) and stiffening constant (β).

Sensor No.	α	β	R^2 (Theoretical)	R^2 (Experimental)
SN1	0.124	0.102	0.9942	0.992
SN2	0.141	0.101	0.9839	0.9874
SN3	0.143	0.118	0.9948	0.9964
SN4	0.125	0.118	0.9996	0.9986
SN5	0.147	0.173	0.9966	0.996
SN6	0.135	0.185	0.9869	0.9846
