# Supplementary material for: Nonlinearity synergy: An elegant strategy for realizing high-sensitivity and wide-linear-range pressure sensing
Source: Nat Commun. 2023 Oct 20;14:6641. doi: 10.1038/s41467-023-42361-9 (PMC10589270; doi:10.1038/s41467-023-42361-9)
Supplement: Supplementary file 3 — Description of Additional Supplementary Files [file 41467_2023_42361_MOESM3_ESM.pdf]

Description of Additional Supplementary Files

Supplementary Movie 1： High-resolution pressure sensing at a highly pressurized state.

Supplementary Movie 2: Grasping test of a tofu block with the aid of a DPyCF@SR sensor.

Supplementary Movie 3： Grasping test of a tofu block with the aid of a Tskscan sensor.

Supplementary Movie 4： Grasping test of a steel block with the aid of a DPyCF@SR sensor.

Supplementary Movie 5： Grasping test of a steel block with the aid of a Tskscan sensor.

Supplementary Movie 6： Sole pressure detection application with a DPyCF@SR sensor.

Supplementary Movie 7： Code-pressure double encryption application with 4 × 4 DPyCF@SR sensor array.
