# Supplementary material for: Nonlinearity synergy: An elegant strategy for realizing high-sensitivity and wide-linear-range pressure sensing
Source: Nat Commun. 2023 Oct 20;14:6641. doi: 10.1038/s41467-023-42361-9 (PMC10589270; doi:10.1038/s41467-023-42361-9)
Supplement: Supplementary file 11 — Reporting Summary [file 41467_2023_42361_MOESM11_ESM.pdf]

nature portfolio

Corresponding author(s): Wei Zhou, Haimin Yao

Last updated by author(s): Sep 29, 2023

Reporting Summary

Nature Portfolio wishes to improve the reproducibility of the work that we publish. This form provides structure for consistency and transparency in reporting. For further information on Nature Portfolio policies, see our Editorial Policies and the Editorial Policy Checklist.

Statistics

For all statistical analyses, confirm that the following items are present in the figure legend, table legend, main text, or Methods section.

n/a	Confirmed	
☐	☒	The exact sample size (*n*) for each experimental group/condition, given as a discrete number and unit of measurement
☐	☒	A statement on whether measurements were taken from distinct samples or whether the same sample was measured repeatedly
☒	☐	The statistical test(s) used AND whether they are one- or two-sided<br>*Only common tests should be described solely by name; describe more complex techniques in the Methods section.*
☒	☐	A description of all covariates tested
☒	☐	A description of any assumptions or corrections, such as tests of normality and adjustment for multiple comparisons
☐	☒	A full description of the statistical parameters including central tendency (e.g. means) or other basic estimates (e.g. regression coefficient) AND variation (e.g. standard deviation) or associated estimates of uncertainty (e.g. confidence intervals)
☒	☐	For null hypothesis testing, the test statistic (e.g. *F*, *t*, *r*) with confidence intervals, effect sizes, degrees of freedom and *P* value noted<br>*Give P values as exact values whenever suitable.*
☒	☐	For Bayesian analysis, information on the choice of priors and Markov chain Monte Carlo settings
☒	☐	For hierarchical and complex designs, identification of the appropriate level for tests and full reporting of outcomes
☒	☐	Estimates of effect sizes (e.g. Cohen's *d*, Pearson's *r*), indicating how they were calculated

Our web collection on statistics for biologists contains articles on many of the points above.

Software and code

Policy information about availability of computer code

Data collection	Digital source meter (Keithley, 2400, USA)
Data analysis	Origin 2021, MATLAB R2019a, Microsoft PowerPoint 2013

For manuscripts utilizing custom algorithms or software that are central to the research but not yet described in published literature, software must be made available to editors and reviewers. We strongly encourage code deposition in a community repository (e.g. GitHub). See the Nature Portfolio guidelines for submitting code & software for further information.

Data

Policy information about availability of data

All manuscripts must include a data availability statement. This statement should provide the following information, where applicable:

- Accession codes, unique identifiers, or web links for publicly available datasets
- A description of any restrictions on data availability
- For clinical datasets or third party data, please ensure that the statement adheres to our policy

Data available in main text and supplementary information, raw data is available on request from the authors because the data set is large and may cause confusion without proper interpretation in public repository.

Research involving human participants, their data, or biological material

Policy information about studies with human participants or human data. See also policy information about sex, gender (identity/presentation), and sexual orientation and race, ethnicity and racism.

Reporting on sex and gender	In our study, we recruited 3 males participated in the physiological signals monitoring and high-resolution pressure sensing study. Gender was not a fact to be considered in the experiment design, where gender is not a variable that affects the results of the experiment. Each volunteer has been paid 200 RMB for the volunteer tests before all the volunteer tests, all volunteers formed consents have been obtained.
Reporting on race, ethnicity, or other socially relevant groupings	N/A
Population characteristics	Three males were involved in the physiological signals monitoring and high-resolution pressure sensing at a highly pressured demonstration, whose ages range from 23-29, weight 60-75 kg.
Recruitment	We posted recruitment flyers in the university to recruit volunteers. The volunteers contacted us by themselves. Self-bias does not have an effect on pulse monitoring and high-resolution pressure sensing experiments.
Ethics oversight	The Medical Ethics Committee of Xiamen University, China

Note that full information on the approval of the study protocol must also be provided in the manuscript.

Field-specific reporting

Please select the one below that is the best fit for your research. If you are not sure, read the appropriate sections before making your selection.

☒ Life sciences ☐ Behavioural & social sciences ☐ Ecological, evolutionary & environmental sciences

For a reference copy of the document with all sections, see nature.com/documents/nr-reporting-summary-flat.pdf

Life sciences study design

All studies must disclose on these points even when the disclosure is negative.

Sample size	3 volunteers with 3 males. All volunteers are healthy in a normal emotion range.
Data exclusions	No data exclusion involved.
Replication	All of the measurements were taken for multiple times. The data at each test location were sampled for multiple times(more than 3 times during the measurements From the measured results, the data showed high similarity, and the reproducibility of all kinds of measurements were good in this study.
Randomization	The human participants were recruited from outside the group by posting flyers.
Blinding	No blinding involved. Blinding is not required for pulse testing and high-resolution pressure experiments.

Reporting for specific materials, systems and methods

We require information from authors about some types of materials, experimental systems and methods used in many studies. Here, indicate whether each material, system or method listed is relevant to your study. If you are not sure if a list item applies to your research, read the appropriate section before selecting a response.

Materials & experimental systems

n/a	Involved in the study	
☒	☐	Antibodies
☒	☐	Eukaryotic cell lines
☒	☐	Palaeontology and archaeology
☒	☐	Animals and other organisms
☒	☐	Clinical data
☒	☐	Dual use research of concern
☒	☐	Plants

Methods

n/a	Involved in the study	
☒	☐	ChIP-seq
☒	☐	Flow cytometry
☒	☐	MRI-based neuroimaging
